# A segmentation-based volumetric approach to localize and quantify cerebral vasospasm based on tomographic imaging data

**DOI:** 10.1371/journal.pone.0172010

**Published:** 2017-02-15

**Authors:** Axel Neulen, Tobias Pantel, Michael Kosterhon, Stefanie Kirschner, Marc A. Brockmann, Sven R. Kantelhardt, Alf Giese, Serge C. Thal

**Affiliations:** 1 Department of Neurosurgery, University Medical Center of Mainz, Mainz, Germany; 2 Department of Neuroradiology, University Medical Center of Mainz, Mainz, Germany; 3 Department of Anesthesiology, University Medical Center of Mainz, Mainz, Germany; Heinrich-Heine-Universitat Dusseldorf, GERMANY

## Abstract

**Introduction:**

Quantification of cerebral vasospasm after subarachnoid hemorrhage (SAH) is crucial in animal studies as well as clinical routine. We have developed a method for computer-based volumetric assessment of intracranial blood vessels from cross-sectional imaging data. Here we demonstrate the quantification of vasospasm from micro computed tomography (micro-CT) data in a rodent SAH model and the transferability of the volumetric approach to clinical data.

**Methods:**

We obtained rodent data by performing an *ex vivo* micro-CT of murine brains after sham surgery or SAH by endovascular filament perforation on day 3 post hemorrhage. Clinical CT angiography (CTA) was performed for diagnostic reasons unrelated to this study. We digitally reconstructed and segmented intracranial vascular trees, followed by calculating volumes of defined vessel segments by standardized protocols using Amira^®^ software.

**Results:**

SAH animals demonstrated significantly smaller vessel diameters compared with sham (MCA: 134.4±26.9μm *vs*.165.0±18.7μm, p<0.05). We could highlight this difference by analyzing vessel volumes of a defined MCA-ICA segment (SAH: 0.044±0.017μl *vs*. sham: 0.07±0.006μl, p<0.001). Analysis of clinical CTA data allowed us to detect and volumetrically quantify vasospasm in a series of 5 SAH patients. Vessel diameters from digital reconstructions correlated well with those measured microscopically (rodent data, correlation coefficient 0.8, p<0.001), or angiographically (clinical data, 0.9, p<0.001).

**Conclusions:**

Our methodological approach provides accurate anatomical reconstructions of intracranial vessels from cross-sectional imaging data. It allows volumetric assessment of entire vessel segments, hereby highlighting vasospasm-induced changes objectively in a murine SAH model. This method could also be a helpful tool for analysis of clinical CTA.

## 1. Introduction

Subarachnoid hemorrhage (SAH), a frequent type of hemorrhagic stroke, is a highly relevant clinical picture in neurointensive care [1–3]. In most cases, SAH results from rupture of an intracranial aneurysm. Besides early brain injury caused by the destructive effects of the bleeding, delayed neurological deterioration due to delayed cerebral ischemia (DCI) occurs in a significant number of patients [4, 5]. DCI after SAH results from processes involving vasospasm of the large cerebral vessels, arteriolar vasospasm, microthrombosis, and ischemia related to cortical spreading depression [4]. It significantly influences neurological outcome, morbidity, and mortality and is therefore in the focus of attention in both clinical and experimental SAH research.

The majority of experimental and clinical studies on SAH and DCI evaluate vasospasm of the large cerebral vessels as an endpoint. Quantification of vasospasm is a major challenge in conducting studies with small animal models of SAH, in which vasospasm is usually analyzed by *ex vivo* assessment of the diameters of arteries forming the circle of Willis in histological sections or after using ink-gelatin vascular casting [6–12]. These methods, however, have the disadvantage that vessel diameters can only be assessed at defined anatomical positions and vasospasm of other vascular segments may be missed. In addition, these techniques are highly dependent on and influenced by the investigator. We presume that a method that allows an anatomically accurate reconstruction of the intracranial vascular tree from cross-sectional imaging data and a subsequent automated analysis of whole vessel segments could overcome these methodological issues. Several studies have described the analysis of intracranial vascular structures in small animals, using vascular casting and micro-computed tomography (micro-CT) [13–17]. However, micro-CT has not been used to analyze vasospasm in experimental SAH studies, because evaluation of resulting cross-sectional data is complicated.

In clinical practice, a timely detection of hemodynamically-relevant angiographic vasospasm is crucial, because this condition can be treated interventionally by endovascular angioplasty or pharmacological vasospasmolysis [1, 2]. Transcranial Doppler sonography is normally used for bedside screening of SAH patients as low velocities of intracranial blood flow practically exclude relevant vasospasm [1–3]. Elevated flow velocities or other clinical signs of vasospasm, on the other hand, frequently require additional imaging [1–3]. For this purpose, most clinical centers use cranial CT angiography (CTA) and perfusion CT (CTP). Clinical studies showed that cranial CTA detects vasospasm of proximal cerebral vessels with high specificity and lower sensitivity [2, 18–21]. However, in mild or moderate cases it remains difficult to estimate the relevance of vasospasm in CTA. We believe that a volumetric method to analyze entire vessel segments could provide a new additional parameter, which may have the potential to estimate the degree of vasospasm depicted by CTA more precisely.

Therefore, our group has set out to develop a method that provides a digital 3-dimensional reconstruction of the intracranial vascular tree based on CTA data and a subsequent automated, segmentation-based evaluation of whole vessel segments. The aim of our study was to validate this method by determining its accuracy concerning digital reproduction of the vasculature and of vessel diameters from *ex vivo* micro-CT of murine brains and from clinical CTA data to test the feasibility to record changes in vessel diameter. Furthermore, we set out to test the feasibility of the method to detect and quantify vasospasm in experimental murine SAH by calculation of the volumes of defined vessel segments and to test the transferability of the volumetric approach to clinical CTA data.

## 2. Materials and methods

### 2.1 Ethics and housing conditions

All procedures involving human participants were approved by the responsible ethics committee (Ethikkommission der Landesärztekammer Rheinland-Pfalz) and were performed in accordance with the 1964 Helsinki declaration and its later amendments. Because anonymized imaging data were analysed and because of the retrospective nature of the study, informed consent was not necessary.

The animal experiments were approved by the responsible animal care committee (*Landesuntersuchungsamt Rheinland-Pfalz*) and carried out in accordance with the German Animal Welfare Act (*TierSchG*). All applicable international, national, and institutional guidelines for the care and use of animals were followed.

The mice were kept under controlled environmental conditions (12 h dark/light cycle, 23 ± 1°C, 55 ± 5% relative humidity), and free access to food (Altromin, Germany) and water.

### 2.2 Experimental SAH model, perfusion casting, and microscopic determination of vascular diameters

**Fig 1:**
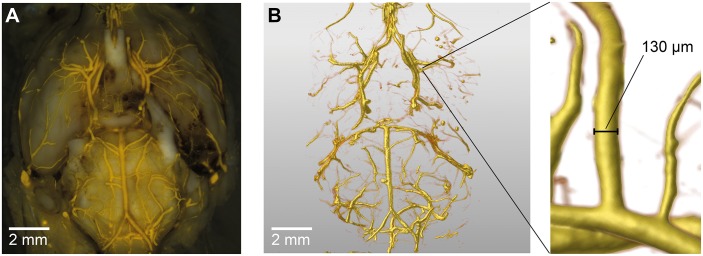
Digital reconstruction of the cerebral vasculature. Representative brain sample (A) with corresponding digital reconstruction of the vasculature (B), determination of a vascular diameter (C).

Experiments were carried out between September and November 2014 at the University Medical Center of Mainz, Germany. SAH was induced in male and female C57BL6 mice (Charles River, Cologne, Germany, age 6 to 9 months) by endovascular filament perforation under general anesthesia, using isoflurane (4 vol% induction, 2 vol% maintenance) as previously described [22]. In brief, after placing an intracranial pressure (ICP) probe (Codman, Johnson & Johnson, Raynham, MA, USA) through a burr hole in the left frontal region, a filament (Prolene 5.0, Ethicon, Norderstedt, Germany) was introduced into the left internal carotid artery and then intracranially advanced, with a rise in ICP as the indicator for successful endovascular perforation, resulting in SAH. SAH was achieved in all animals of the SAH group as verified by subarachnoid hematoma in the SAH specimens as shown exemplarily in Fig 1. We monitored body temperature and maintained it at 37°C with a heating pad throughout the surgical procedure. At the end of surgery, we removed the ICP probe and placed the animals inside an incubator heated to 33°C at a humidity of 35% (IC8000, Draeger, Germany) to prevent hypothermia as described [23]. Additionally, we performed identical surgery in sham animals, this time, without endovascular perforation. Weight was monitored daily in mice with SAH (day 0: 31.8±5.3 g; day 1: 30.2±5.8; day 2: 30.1±6.6 g; day 3: 27.6±6.5g) and in sham animals (day 0: 31.9±5.7g; day 1: 31.0 ±6.7g; day 2: 30.3±7.2g; day 3: 26.7±6.7g).

Of 13 SAH and 9 sham animals, 7 animals died before undergoing transcardial perfusion (SAH: 1 animal died intraoperatively, 2 were found dead on postop. day 2, 1 on day 3; sham: 3 animals were found dead on day 3). Surviving animals (9 SAH, 6 sham animals), a group size that is similar to other studies on murine cerebral vasospasm [6–12], were subjected to transcardial perfusion and endovascular casting [13–17] 3 days after induction of SAH. In detail, we induced anesthesia by intraperitoneally injecting 5 μg/g body weight (bw) midazolam (Ratiopharm, Ulm, Germany), 30 ng/g bw fentanyl (Curamed, Karlsruhe, Germany), and 0.5 μg/g bw medetomidin (Pfizer, Karlsruhe, Germany). After reaching sufficiently deep anesthesia (no reaction to painful stimuli) we punctured the left ventricle, using a 21G cannula (B Braun Melsungen AG, Melsungen, Germany), then opened the right atrium and transcardially perfused the animals for 2 minutes with Dulbecco’s Phosphate Buffered Saline containing MgCl_2_ and CaCl_2_, at pH 7.4 (Sigma-Aldrich, Hamburg, Germany). We continued this procedure for 4 minutes with a 4% paraformaldehyde solution (Sigma-Aldrich), while keeping the solution temperatures at 37°C (98.6°F). We monitored the perfusion pressure by using a riser tube, keeping it constantly at 70±10 mmHg during perfusion as previously described [11]. After perfusion with PFA, we clamped both the descending aorta and the inferior vena cava and continued with transcardial perfusion for 20 min, using Microfil^®^ MV-122 (Flowtech Inc., Carver, MA, USA) at a constant rate of 0.2 ml/min. To cure the radiopaque casting agent, the cadavers were stored at room temperature for 2 hours and then decapitated. Subsequently, we removed the skin and jaws and decalcified the skulls by incubating these in 8% formic acid (Sigma-Aldrich) at room temperature for 48 hours. Afterwards, we carefully removed the skull base while maintaining the convexity of the skull and transferred the preparations to a 4% PFA solution to be stored at 4°C.

### 2.3 Microscopic determination of vessel diameters

**Fig 2:**
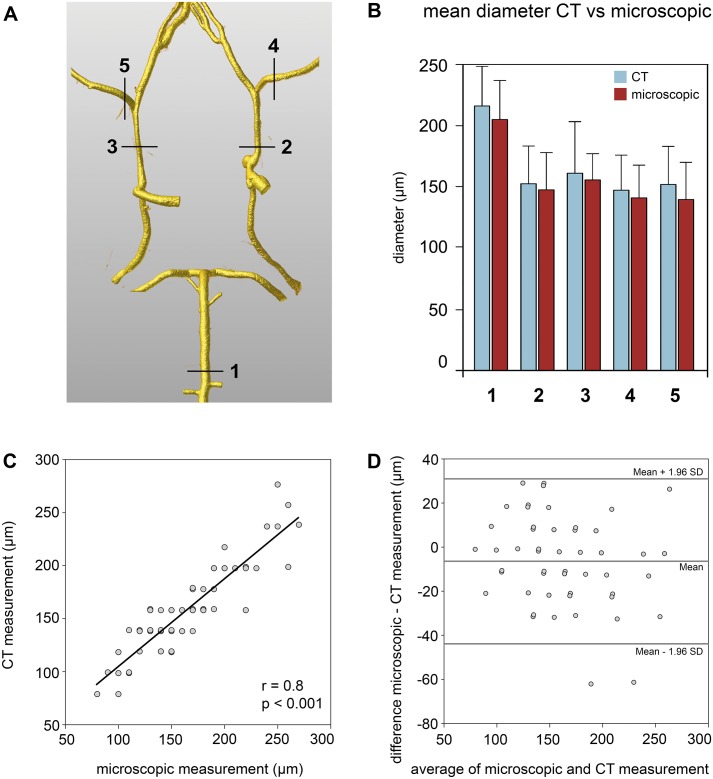
Accuracy of digital reconstruction of the murine cerebral vasculature from micro-CT. (A) Anatomical points, at which vascular diameters were determined. (B) Mean diameters measured from the digitally reconstructed vasculature (blue) and microscopically from brain samples (red). (C) Correlation of digital and microscopic measurements. (D) Bland-Altman-Plot comparing digital and microscopic measurements.

We assessed the diameters of the basal vessels with a computerized image system: a high-resolution camera (Infinity X-21, Deltapix, Maalov, Denmark) with DeltaPix Insight software version 2.0.1 (Deltapix). The software was calibrated to a micrometer measuring scale. Thus, we were able to determine vascular diameters in the following regions: middle of basilar artery, left and right internal carotid artery (ICA), and left and right middle cerebral artery (MCA, see Figs 1 and 2).

### 2.4 Micro-computed tomography and 3-dimensional reconstruction of the vascular tree from micro-CT data

For this purpose, we used an industrial micro-CT system (Y.Fox, Yxlon, Garbsen, Germany), in which the object is rotated around its horizontal axis. The micro-CT is equipped with a multifocus transmission x-ray tube (10 to 160 kV; focal spot sizes 1.3 μm and 5 μm), a CNC manipulator, which can rotate and move objects in micrometer scale and a 14-bit direct amorphous silicon flat panel detector (Varian PaxScan 2520 D/CL; Varian, Palo Alto, CA, USA) [24, 25].

Using a tube voltage of 80 kV, the tube current was set to 38 μA to avoid overexposure of the detector. A step-and-shoot image acquisition protocol was used. Images were acquired at 10 fps. To optimize the signal-to-noise ratio (SNR), exposure time for each projection was set to 1 s [26]. A total of 1000 projections were acquired per scan with an angle increment of 0.36°/projection (360°/1000 images). A filtered back projection algorithm applying the Shepp-Logan filter with a matrix of 1024x1024x1024 voxels was used for reconstruction of RAW-Data (Reconstruction Studio, Tera Recon), resulting in a voxel size of 15 μm.

We then imported Dicom datasets into the Amira^®^ software version 5.4.2 (FEI Visualization Sciences Group, Hillsboro, OR, USA). The vascular tree was visualized, using the function *Volren* with a visualization threshold of 180 arbitrary units (Fig 1B and 1C).

### 2.5 Clinical cranial computed tomography angiography and 3-dimensional reconstruction of the vascular tree from clinical CTA data

The clinical CTAs and digital subtraction angiographies (DSAs) were performed according to diagnostic standard protocols for diagnostic reasons unrelated to the present study. We retrospectively identified DSA data sets that were generated within 90 minutes after a CTA data set in patients with the diagnosis of SAH. These imaging data were anonymized and exported as DICOM files for evaluation.

Afterwards, we imported resulting DICOM data into the Amira^®^ software, and digitally substracted radiopaque bony structures. The vascular tree was visualized, using the function *Volren*. The visualization threshold was determined by windowing, so that the large bridge veins were depicted in sharp outlines. For better comparability with angiography data, we finally visualized the data, using the *virtually reconstructed radiograph function* of *Volren*.

### 2.6 Segmentation of the virtual vascular tree, color-coding of vascular diameters, and volumetric analysis of defined vascular segments

**Fig 3:**
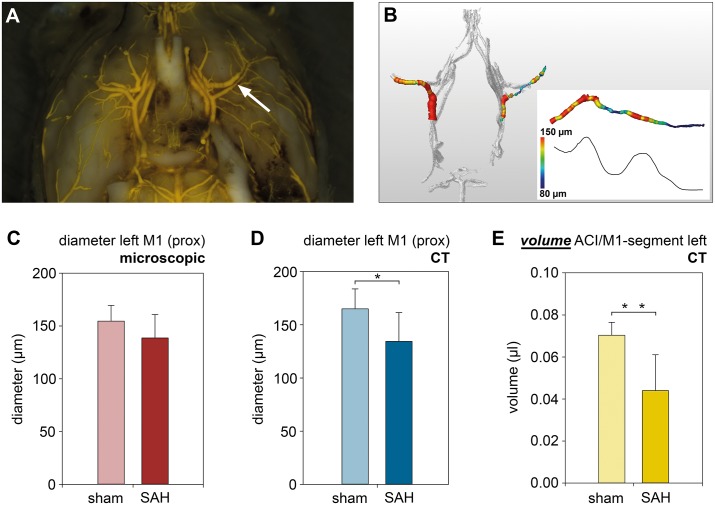
Volumetric evaluation of vascular segments to quantify vasospasm. (A) Murine SAH brain sample with vasospasm of the left MCA (white arrow) with (B) corresponding digitally reconstructed cerebral vasculature with color-coded representation of the course of the vascular diameter.(C) Diameters for sham and SAH mice at the (proximal) M1 segment determined microscopically. (D) Diameters for sham and SAH mice at the (proximal) M1 segment determined from the digitally reconstructed vascular trees. (E) Volumetric evaluations of the left ACI-MCA segment for sham and SAH mice. Note that the volumetric method reveals larger and highly significant differences between sham and SAH compared with the measurements derived from vascular diameters.

**Fig 4:**
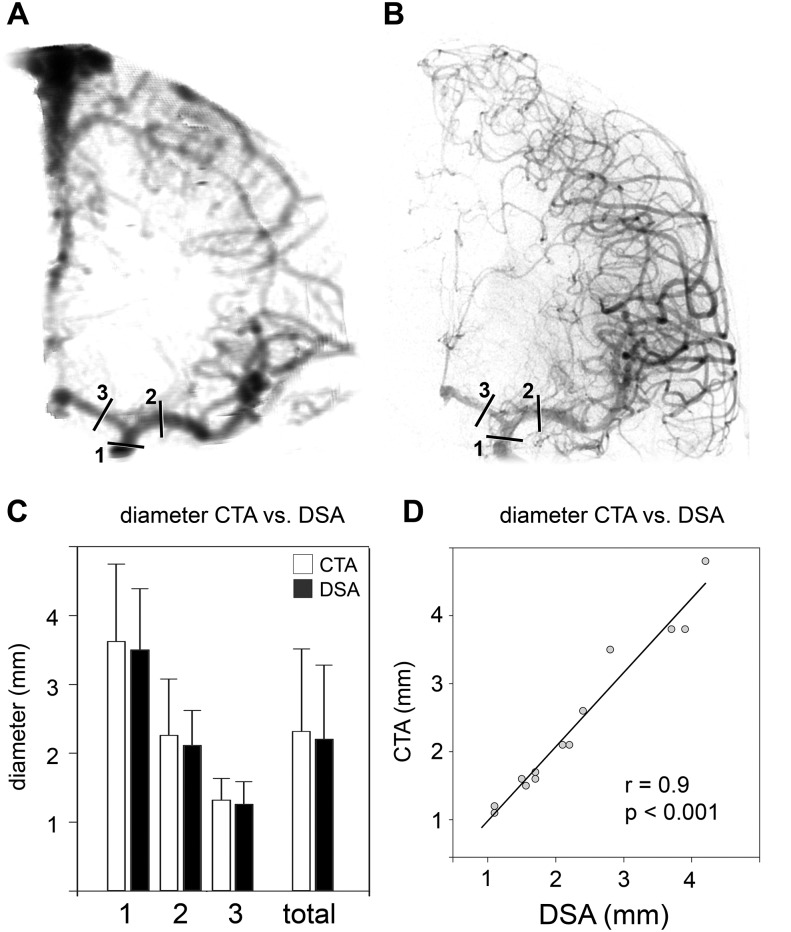
Accuracy of digital reconstruction of the cerebral vasculature from clinical CT angiography. Vascular diameters of ICA, ACA, and MCA were determined from CTAs (A) and from corresponding digital subtraction angiographies (B). (C) Mean diameters measured from digitally reconstructed CTAs (white) and from angiographies (black). (D) Correlation of vessel diameters determined from digitally reconstructed CTAs and angiographies.

We virtually dissected the vessels of the circle of Willis by using the function *VolumeEdit*. Thus, we were able to determine the diameters of the basal arteries at anatomically defined points (Fig 2A–2C), using a virtual 3-dimensional ruler—a subfunction of the *Measurement Function*. We then calculated the vascular center lines, using the function *SpatialGraph* (threshold set at visualization threshold) with subsequent autosegmentation of the vessels into subsegments of the thickness of one voxel. To evaluate micro-CT data sets, we identified a vascular segment consisting of 1 mm internal carotid artery (ICA) and 2.5 mm middle cerebral artery (MCA) and calculated its volume, using the function *SpatialGraphStatistics*, by addition of the volumes calculated for the voxel-sized subsegments, based on a cylindrical model. Moreover, we visualized the subsegments of the 3.5 mm vascular segments with colors reflecting the diameter of the vascular subsegments (Fig 3B). For clinical CTA data representative vascular segments of ICA, ACA, and MCA were analyzed (Fig 4).

### 2.7 Statistics

For statistical evaluation, we used the software Sigma Plot version 12.5 (Systat Software Inc., San Jose, CA, USA). Data are presented as mean ± standard deviation. A student’s unpaired t-test was used to test the statistical significance, with p < 0.05 considered as statistically significant. The t-test sample size function (alpha 0.05, power of 0.8) was used for power analysis to calculate sample sizes. Correlation analyses were calculated with Spearman’s correlation coefficients.

## 3. Results

### 3.1 Accuracy of the 3-dimensional reconstruction of the virtual vascular tree based on micro-CT data

Our 3-dimensional vascular models based on micro-CT data resulted in a highly accurate vascular anatomy in all samples (see Fig 1). We did not detect any discrepancies concerning the anatomy of the circle of Willis between the evaluations with microscopy and those based on micro-CT data. The vessels of the circle of Willis were contrasted by the casting agent in all samples, as well as their vascular branches. To analyze accuracy, we determined the vessel diameters from the digital reconstructions and compared these with the diameters revealed by microscopic analysis of the brain samples. In detail, we determined vascular diameters at five anatomically defined points depicted in Fig 2A in all 15 samples (9 SAH animals, 6 sham animals). Due to an air bubble trapped in the intravascular cast we excluded 1 measurement of the left ICA, and 1 of the left MCA, both in SAH samples. Hence, we obtained 73 data sets with diameters obtained either microscopically or digitally from micro-CT (Fig 2). The barplot depicted in Fig 2B shows similar vessel diameters at all five anatomical positions for the micro-CT reconstruction model and those diameters determined microscopically, with a non-significant trend towards larger diameters in micro-CT data. The CT to microscopic diameter ratio was 1.03±0.2 (n = 73). These data were confirmed by a highly positive correlation between the diameters determined visually and digitally (n = 73; correlation coefficient 0.8, p<0.001, Fig 2C). The Bland-Altman analysis comparing digitally reconstructed diameters with visually determined diameters, confirmed a high correlation and agreement of both methods (Fig 2D).

### 3.2 Volumetric analysis of defined vessel segments and quantification of vasospasm in the murine SAH model

We assessed vasospasm in mice with SAH to investigate the suitability of digital analysis of cross-sectional data to quantify vasospasm in experimental small animal models. Microscopic determination of the diameter of the MCA distal of the carotid T showed smaller diameters in SAH mice compared with control animals (left MCA: 132.1±29.8 μm (SAH, n = 8) vs. 154.6±14.8 μm (sham, n = 6); right MCA: 129.9±28.2 μm (SAH, n = 9) vs. 154.6±23.9 μm (sham, n = 6), see Fig 3C). Moreover, measurement of vascular diameters using the digitally reconstructed vascular tree, revealed similar diameters (left MCA: 134.4±26.9 μm (SAH, n = 8) vs. 165.0±18.7 μm (sham, n = 6), p<0.05; right MCA: 141.1±31.8 μm (SAH, n = 9) vs. 168.3±17.7 μm (sham, n = 6), see Fig 3D). The vessel diameters determined at the MCA correlated well between microscopy and micro-CT evaluation (0.80, p<0.001), which shows an accurate reproduction of vessel diameters. In a next step, we determined volumes of vascular segments consisting of 2.5 mm MCA and 1 mm ACI. As a result of the quantification technique, the differences in vessel volumes between SAH and sham mice was markedly larger compared to the simple measurements of vessel diameters (left MCA: 0.044±0.017 μl (SAH, n = 8) vs 0.07±0.006 μl (sham, n = 6), p<0.001; right MCA: 0.046±0.013 μl (SAH, n = 9) vs. 0.073±0.005 μl (sham, n = 6), p<0.001, Fig 3E), demonstrating vasospasm in the SAH group.

### 3.3 Calculation of animal numbers for future studies

As the changes in volumetric parameters were larger than those in diameter, we set out to perform a power analysis to find out whether the application of the volumetric technique has the potential to reduce animal numbers in future studies. For this purpose, we conducted a hypothetic treatment study with 2 SAH groups. The aim of this study was to gain statistical significance for an improvement of 80% in the respective parameter based on the difference between the control group and the SAH group, assuming the standard deviation of the SAH group of the present study.

Using the data from either the left ACI-M1 segment or the left M1 diameter, we found that we would need a number of 45 animals to analyze changes in diameter, whereas to analyze changes in vascular volume we would only need a minimum of 13 animals. The same is true when using data from either the right ACI-M1 segment or the right M1 diameter. In this case, we identified a number of 33 animals to analyze changes in diameter and only 7 animals to analyze changes in vascular volume.

### 3.4 Accuracy of the 3-dimensional reconstruction of the virtual vascular tree and volumetric analysis of vasospasm from clinical CTA data

We analyzed data from 6 cranial CT-angiographies derived from 5 patients with SAH and corresponding digital subtraction angiographies performed within 23 to 64 minutes after the CT angiography either for aneurysm or interventional vasospasm treatment. As with micro-CT data, the vascular anatomy was depicted clearly. In analogy to the murine data, we analyzed accuracy by determining the vessel diameters of ICA, MCA, and ACA from the digitally reconstructed CTAs and compared these with the diameters obtained from the angiographies (Fig 4). The bar graph depicted in Fig 4C shows similar vessel diameters at all three anatomical positions for the CTA reconstruction and those diameters determined angiographically. These data show a high positive correlation between the diameters determined from angiography or from CTA (correlation coefficient 0.9, p<0.001, Fig 4D), indicating a precise reproduction of vessel diameters.

**Fig 5:**
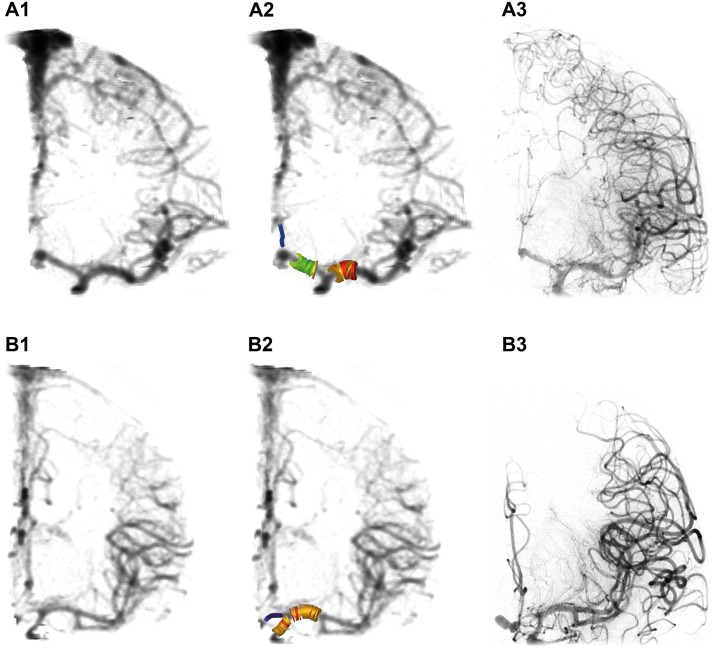
Analysis of vasospasm from clinical CTA data. Cranial CTA data of 2 SAH patients (A, B), shown as virtually reconstructed radiograph without (A1, B1) and with (A2, B2) color-coding of representative segments of ICA, MCA, and ACA. Note the vasospasm of A3 segment (patient A) and A1 segment of the ACA (patient B). A3, B3 show the respective digital subtraction angiographies.

We set out to assess whether a volumetric evaluation would also highlight vasospasm-induced changes due to larger changes in vessel volumes compared to vessel diameters in the clinical series. For this purpose, we measured the vessel diameters and volumes of the M1 segment and of the ICA. The vessel diameters of the M1 segments were 2.1±0.8 mm, and of the ICA 3.6±1.0 mm. In contrast, the vessel volumes of the M1 segments were 1.4±1.0 μl/mm, and of the ICA 5.6±3.4 μl/mm, indicating that the volumetric evaluation of entire vessel segments reacts more sensitive than the reduction in diameter. Two cases are exemplarily illustrated in Fig 5.

## 4. Discussion

In the present study we demonstrate a novel method to calculate vessel volumes of entire vessel segments from cross-sectional imaging data. This technique allows for the first time, to the best of our knowledge, to quantify cerebral vasospasm from micro-CT data in *eg* rodent SAH models. Furthermore, we demonstrate the transferability of the method to clinical CTA data. The volumetric assessment is advantageous for examining vasospasm of large cerebral vessels, because a reduction of the vessel diameter induces a greater reduction of the volume of the affected vascular segment.

DCI is a major factor contributing to neurological outcome in SAH patients. DCI results from several processes associated with SAH; besides vasospasm of large cerebral vessels and arteriolar vasospasm, other factors including microthrombosis and cortical spreading ischemia [reviewed in [4]] are thought to play a major role. A limitation of the present method presented here is that it can only be used to quantify vasospasm of cerebral vessels, but does not allow conclusions concerning other factors leading to DCI. More pathophysiological information could be provided in future experimental studies by *in vivo* micro-CTP and *in vivo* micro-CTA in murine and other rodent models of SAH, as CTP is well established in this context in clinical practice [27–30]. A recent study performed *in vivo* high resolution digital subtraction angiography [31] and cerebral CTA of the murine cerebrovasculature [25]. These procedures, however, have not yet been tested in mice with SAH-related vasospasm. CTP, to our knowledge, has not yet been used so far in small animal models. Nevertheless, vasospasm of the large cerebral vessels is investigated as a key endpoint in many experimental and clinical studies on SAH and DCI. Our method provides a new, valuable tool for quantification of vasospasm from rodent microCT data in experimental studies.

Other small animal studies established vascular casts of the cerebrovasculature to analyze cerebrovascular anatomy by using micro-CT [13–17]. Based on this finding, the present study demonstrates a novel method by using Amira^®^ software, reconstructing the intracranial vascular tree 3-dimensionally from digital cross-sectional data, and allowing us to analyze diameters and volumes of defined vessel segments. The method’s high accuracy is demonstrated by comparing vascular diameters obtained microscopically from anatomical preparations with digitally reconstructed vascular trees. The method used in our study has the advantage of evaluating entire vessel segments, presumably reflecting the degree of vasospasm more accurately than vessel diameters at single points. By using algorithms of Amira^®^ software, we are able to calculate diameters and volumes of voxel-sized subsegments of selected vessels. This enables us to calculate the volumes of defined vascular segments of interest by addition of the volumes of the voxel-sized subsegments. Color-coding the diameters of the subsegments may be a helpful and intuitive tool to visualize vasospasms as shown in Figs 3 and 5. As this method is based on Amira^®^ software algorithms, its investigator dependence is low.

Analysis of the vessel volumes of a defined MCA-ICA segment detected significantly lower vessel volumes in SAH compared with control animals, showing evidence of vasospasm. In line with this, we found that the diameters of the proximal M1 segments measured microscopically or derived from the digitally reconstructed vascular tree, were markedly smaller in the SAH group. Considering the vessel as a more or less round body, the larger differences with vascular volumes compared to vascular diameters are simply explained by the fact that the square of diameters is included into volume calculation. An advantage of measuring the greater reduction of the vessel volume is that it may reduce animal numbers needed in future experimental treatment studies. Whether the putatively higher sensitivity of volume reduction could also be helpful in the clinical setting needs to be examined in future clinical studies, correlating volumetric evaluation with neurological status of the patient and CT perfusion measurements.

Other authors reported vasospasm-related vessel narrowing by 21% [8] and 20% [9] at day 3 post hemorrhage in murine SAH models based on injection of autologous blood into the cisterna magna [8] or endovascular perforation [9], which is in a similar range as our results. These studies performed microscopic evaluations of the circle of Willis following injection of casting agents based on ink and gelatin after perfusion fixation, which is similar to the casting procedure used in our study. Other studies with murine SAH models described apparently larger degrees of vasospasm-related reduction of vessel size by 27% [6] and 34% [7]. However, these studies performed measurements of the circumference of the basilar artery [6] and the cross sectional area of the basilar artery in histological sections [7]. These data confirm our findings, because we found similar reductions in vascular volumes, which correlate with circumference and cross sectional area of the vessel, and indicate, that perfusion-casting procedures yield similar degrees of vasospasm as histological techniques.

The method is not limited to analysis of vasospasm in experimental small animal models, but can also be applied to clinical patient computed tomography angiography (CTA) data, as depicted in Figs 4 and 5. We found a high correlation between vessel diameters determined by means of angiography and reconstructed CTA, demonstrating the applicability to clinical CTA data. This is in line with other studies, which showed that CTA can be used for detection of vasospasm of the large cerebral vessels with an accuracy comparable to that of DSA [20], and has a high specificity but lower sensitivity in detection of location and severity of posthemorrhagic vasospasm [20, 21]. The method presented in this paper could be useful to analyze the course of vasospasm in patients. In addition, volumetric evaluation of entire vessel segments may provide an additional parameter to estimate the relevance of vasospasm depicted by CTA. However, further studies are needed to verify the potential of this method.

## Abbreviations

ACA: anterior cerebral artery
CTA: computed tomography angiography
DCI: delayed cerebral ischemia
DICOM: Digital Imaging and Communications in Medicine
ICA: internal carotid artery
MCA: middle cerebral artery
micro-CT: micro-computed tomography
SAH: subarachnoid hemorrhage
